# A dynamic nomogram for predicting intraoperative brain bulge during decompressive craniectomy in patients with traumatic brain injury: a retrospective study

**DOI:** 10.1097/JS9.0000000000000892

**Published:** 2023-12-02

**Authors:** Dongzhou Zhuang, Tian Li, Huan Xie, Jiangtao Sheng, Xiaoxuan Chen, Xiaoning Li, Kangsheng Li, Weiqiang Chen, Shousen Wang

**Affiliations:** a Department of Neurosurgery, Fuzong Clinical Medical College of Fujian Medical University, Fuzhou; b Department of Microbes and Immunity, Shantou University Medical College, Shantou, Guangdong; c Department of Neurosurgery, First Affiliated Hospital, Shantou University Medical College, Shantou, Guangdong; d Department of Orthopaedics, The Third Xiangya Hospital, Central South University, Changsha, Hunan, People’s Republic of China

**Keywords:** decompressive craniectomy, intraoperative brain bulge, machine learning, nomogram, traumatic brain injury

## Abstract

**Objective:**

The aim of this paper is to investigate the risk factors associated with intraoperative brain bulge (IOBB), especially the computed tomography (CT) value of the diseased lateral transverse sinus, and to develop a reliable predictive model to alert neurosurgeons to the possibility of IOBB.

**Methods:**

A retrospective analysis was performed on 937 patients undergoing traumatic decompressive craniectomy. A total of 644 patients from Fuzong Clinical Medical College of Fujian Medical University were included in the development cohort, and 293 patients from the First Affiliated Hospital of Shantou University Medical College were included in the external validation cohort. Univariate and multifactorial logistic regression analyses identified independent risk factors associated with IOBB. The logistic regression models consisted of independent risk factors, and receiver operating characteristic curves, calibration, and decision curve analyses were used to assess the performance of the models. Various machine learning models were used to compare with the logistic regression model and analyze the importance of the factors, which were eventually jointly developed into a dynamic nomogram for predicting IOBB and published online in the form of a simple calculator.

**Results:**

IOBB occurred in 93/644 (14.4%) patients in the developmental cohort and 47/293 (16.0%) in the validation cohort. Univariate and multifactorial regression analyses showed that age, subdural hematoma, contralateral fracture, brain contusion, and CT value of the diseased lateral transverse sinus were associated with IOBB. A logistic regression model (full model) consisting of the above risk factors had excellent predictive power in both the development cohort [area under the curve (AUC)=0.930] and the validation cohort (AUC=0.913). Among the four machine learning models, the AdaBoost model showed the best predictive value (AUC=0.998). Factors in the AdaBoost model were ranked by importance and combined with the full model to create a dynamic nomogram for clinical application, which was published online as a practical and easy-to-use calculator.

**Conclusions:**

The CT value of the diseased lateral transverse is an independent risk factor and a reliable predictor of IOBB. The online dynamic nomogram formed by combining logistic regression analysis models and machine learning models can more accurately predict the possibility of IOBBs in patients undergoing traumatic decompressive craniectomy.

## Introduction

Highlights

- The effective prediction model and clinical application tools that are not available at present are proposed.
- The combination of the machine learning model and logistic regression model improved the validity and accuracy of the model.
- It is proposed for the first time that computed tomography values of diseased lateral transverse sinus are highly correlated with the occurrence of intraoperative brain bulge (IOBB) in decompressive craniectomy surgery of patients with traumatic brain injury.
- The results suggest that the venous system plays an important role in the occurrence of IOBB.

Traumatic brain injury (TBI) is a common neurosurgical condition. According to the World Health Organization, the number of people who die from TBI is ~100 000–120 000 worldwide each year, and the number of people who suffer minor or moderate TBI are in the millions or even tens of millions^1^. After TBI, decompressive craniectomy (DC) is recommended when the patient has a decreased level of consciousness, an increasing hematoma, a midline shift, or a basal sink^2,3^. During DC surgery, brain tissue sometimes expands and swells rapidly in just a few minutes and protrudes the bony window. This phenomenon is called intraoperative brain bulge (IOBB)^4^. It is also known as intraoperative brain herniation and intraoperative encephalocele. Upon onset, the disease develops rapidly and can cause ischemic necrosis of brain tissue and even death^5^.

Although IOBB has received increasing attention from neurosurgeons, its mechanism is complex and unknown. Currently, several theories have been proposed based on clinical studies. The main mechanisms include delayed intracranial hematoma or distal septal site hematoma, acute diffuse brain swelling, reperfusion injury, and impaired venous return^6–8^. Delayed hematomas can be resolved by reoperation, whereas other causes of IOBB are more complex and fatal. During DC surgery, after removing the bone flap and cutting the dura mater, the intracranial pressure (ICP) is released, and there is a time difference between arteries and veins in the process of blood flow restoration; the arteries are immediately congested while the veins or the microcirculatory reflux is blocked, the brain tissue is congested with blood and rapidly swelling, which may be one of the reasons for the occurrence of IOBB^5,9,10^. Therefore, an increasing number of studies have focused on the alterations of the venous system during IOBB.

In clinical practice, when a patient with TBI requires DC surgery, it often means that the patient’s condition is critical and urgent^11^. At this time, computed tomography (CT) is commonly used instead of computed tomography angiography (CTA) and computed tomography venography (CTV), creating a challenge to assess the status of the venous system by CT^12^. Previous studies have shown that high-density shadowing of venous sinuses is closely associated with venous sinus thrombosis and also with venous reflux velocity^13^. This suggests that CT values of the venous sinus may be representative of the state of the venous system^14^. One of the objectives of this study was to investigate the relationship between the CT values of venous sinus and IOBB, thus supporting the idea that venous return obstruction is an important factor in the development of IOBB. In addition, although there have been a few case reports and risk factor studies in recent years^3,15,16^, neurosurgeons are interested in accurately predicting the occurrence of IOBB before surgery and avoiding this urgent and dangerous situation. Therefore, another objective of this study was to investigate the risk factors associated with the occurrence of IOBB in patients with TBI and to develop an accurate and effective prediction model combining basic clinical characteristics, hematology, and imaging.

## Patients and methods

### Patient population

The patients included in this study were all TBI patients requiring standard surgery of DC. All patients were treated according to the guidelines for Traumatic Brain Injury surgery of the American Association of Neurosurgeons^17^, including medical treatment and indications for DC surgery. According to the guidelines for Traumatic brain Injury surgery of the American Association of Neurosurgeons^17^, DC surgery is a life-saving operation for patients with acute TBI and malignant intracranial hypertension who failed to respond to medical treatment. The indications for surgery include progressive disorder of clinicians, significant mass effect of intracranial injury on CT scan, increased ICP greater than 30 mmHg, and ineffective medical treatment such as dehydration or even dilated pupils in patients with acute TBI^18^. The inclusion criteria for the study were as follows: (1) TBI patients were older than 18 years and (2) emergency DC was needed after admission. Patients having the following were excluded: (1) brain swelling due to hypoxia or hypotension, (2) coagulation disorders or taking antiplatelet or anticoagulant medications, (3) preoperative Glasgow Coma Scale (GCS) score of 3 with no improvement after treatment in the emergency room, (4) unstable vital signs unable to tolerate surgery, (5) TBI combined with severe damage to other areas, and (6) preoperative test results not available from other hospitals.

In the developmental cohort, this study reviewed 2451 patients with TBI who were treated at Fuzong Clinical Medical College of Fujian Medical University between 1 January 2015 and 30 September 2021, and a total of 753 patients with TBI with indications for DC surgery were collected according to the indications for DC surgery as elaborated above. According to the exclusion criteria, 644 TBI patients who underwent DC surgery were finally included. With the same screening criteria, the external validation cohort covered 293 TBI patients who underwent DC surgery at the First Affiliated Hospital of Shantou University Medical College between 18 December 2020 and 1 May 2022 (Supplementary Fig. S1, Supplemental Digital Content 2, http://links.lww.com/JS9/B448). This study was approved by the Fuzong Clinical Medical College of Fujian Medical University (No: 2023-045) and the First Affiliated Hospital of Shantou University Medical College (No: B-2022-004) and was conducted in accordance with the Declaration of Helsinki. As this was a retrospective study, the ethics committee approved the waiver of signing the informed consent form. This study has been reported in accordance with STROCSS standards^19^ (Supplemental Digital Content 1, http://links.lww.com/JS9/B447).

### Definition and diagnostic criteria of IOBB

The diagnosis and definition of IOBB in this study were based on the phenomena observed by the surgeon intraoperatively and recorded in the operative record; if it was not recorded in the operative record, it needed to be further determined in conjunction with the postoperative review of the images. Definition and diagnostic criteria of IOBB: (1) intraoperative DC with brain tissue bulging more than 1 cm toward the inner edge of the bone window, brain tissue significantly compressed by the edge of the bone window, brain pulses significantly weakened or unreachable, and brain tissue unable to be reduced; (2) brain tissue bulging <1 cm from the inner edge of the bone window, but progressive bulging, with repeat CT suggestive of a delayed hematoma, a new large cerebral infarction, or diffuse cerebral edema; (3) active dehydration and/or hyperventilation unable to be relieved; (4) exclusion of brain tissue entrapment due to small bone window and protrusion of brain tissue due to change in position^20,21^.

### Data collection

A total of 937 patients who met the enrollment criteria were included in this study. The development cohort included 644 patients, and the validation cohort included 293 patients. Demographic and clinical variables were collected upon admission, including age, gender, GCS score at admission, mechanism of injury, coagulation, pupil condition, surgical approach, preoperative time, operative time, and glucose level. Patients were classified as light (13–15 points), medium (9–12 points), and severe (3–8 points) according to their GCS scores at admission.

Imaging variables obtained from the admission CT included the site of cerebral contusion, unilateral and bilateral injuries, contralateral fractures, degree of midline shift, condition of the basal pool, multiple hematomas, intraventricular hemorrhage (IVH), subarachnoid hemorrhage (SAH), subdural hemorrhage (SDH), and epidural hemorrhage (EDH). We evaluated CT measurements of the venous sinus using Hensfeld Units (HU). The CT values of the [torcular herophili (TH)], superior sagittal sinus (SSS), healthy lateral transverse sinus (HLTS), and diseased lateral transverse sinus (DLTS) acquired on CT are shown in Supplementary Figure S2 (Supplemental Digital Content 3, http://links.lww.com/JS9/B449). CT values were obtained by two neurosurgeons with 10 years of experience each, who independently performed qualitative image analysis without any clinical information about the patients. They were asked to measure their CT values by tracing regions of interest at four different sites: the superior sagittal sinus was evaluated in coronal reconstruction at the level of the posterior border of the third ventricle; the bilateral transverse sinuses were evaluated in sagittal reconstruction at the median site^22^. The torcular herophili was assessed at the confluence of the posterior superior sagittal and transverse sinuses. In each region, they collectively randomly measured CT values at three different points that could be reliably distinguished from the surrounding brain parenchyma, and the mean values were recorded^23^.

### Statistical analysis

All statistical analyses were performed using Statistical Product and Service Solutions (26th version; IBM Corporation, Armonk, New York, USA) and R (version 4.1.0; R Foundation, Vienna, Austria) with appropriate packages (R Foundation for Statistical Computing)^24–26^. Continuous data variables were expressed as the median and interquartile range (IQR), and categorical variables were expressed as counts and percentages. We analyzed between-group differences to reflect differences in patient demographic, clinical, and imaging characteristics. To prevent bias, 1:1 matching was performed using Propensity Score Matching (PSM) based on GCS scores. All parameters showing a statistical trend (*P*<0.05) in univariate analysis were included in a multivariate regression model to identify parameters being independently associated with IOBB. Also, commonly used or reported indicators such as age, sex, and glucose were included in the multivariate regression analysis. Independent risk factors of IOBB with the forward selection procedure, retaining variables with *P*<0.05, were selected for multivariate regression analysis.

The model for predicting IOBB was based on multivariate logistic regression: the basic model included age, SDH, contralateral fracture, and brain contusion as independent risk factors. The full model included the basic model and CT value of DLTS. The area under the receiver operating characteristic (ROC) curve was used to judge and compare the predictive ability of the two models^27^. Decision curve analysis (DCA) was used to assess the value of the clinical application of the models and to compare the net benefits of the models^28^. Net reclassification improvement (NRI) and integrated discrimination improvement (IDI) were used to compare the predictive power of the two models^29^. The external validation of this study was performed using a temporal validation method, collecting data from similar patients in different time periods and different hospitals for further testing of the predictive model established in the development cohort^30^.

Several machine learning models, including Decision Tree (DT), Support Vector Machine (SVM), Random Forest (RF), and AdaBoost, were compared with traditional logistic regression models to find the optimal prediction model. The ROC curves between the different methods were compared using the Delong test^31^. The ranking of the importance of the hazard factors clearly showed the weights of each factor. By combining the optimal prediction model and the importance of the factors, a new nomogram was constructed to predict the possibility of IOBB in DC surgery. To facilitate the application of the prediction model, we upgraded the nomogram to a dynamic nomogram and uploaded it to the shinyapps.io platform (https://dzzhuang.shinyapps.io/dynnomapp/)^30^.

## Results

### Characteristics of the patients in the development cohort

A total of 937 patients were included in the final analysis, including 644 in the development cohort and 293 in the validation cohort (Supplementary Fig. S1, Supplemental Digital Content 2, http://links.lww.com/JS9/B448). Of the 644 patients in the development cohort, 93 (14.4%) developed IOBB during the DC procedure (Table 1). Patients who developed IOBB were younger than those who did not develop IOBB. Patients who developed IOBB all had a severe cranial injury (GCS ≤8 points) and had a higher probability of bilateral pupillary dilatation (45.2% vs. 19.2%) and mortality than those who did not develop IOBB (81.7% vs. 19.4%).

**Table 1:** Differences between with no IOBB group and the IOBB group in the development cohort.

Characteristic	No IOBB (*n*=551)	IOBB (*n*=93)	*P*
Gender (male)	431 (78.2%)	74 (79.6%)	1.000
Age	58 (49, 67)	48 (31, 60)	<0.001
GCS grade			<0.001
Mild (13–15 score)	44 (8%)	0 (0%)	
Moderate (9–12 score)	76 (13.8%)	0 (0%)	
Severe (3–8 score)	431 (78.2%)	93 (100%)	
Mechanism of injury			0.080
Car accident	343 (62.3%)	68 (73.1%)	
High fall injury	181 (32.8%)	20 (21.5%)	
Other	27 (4.9%)	5 (5.4%)	
Coagulation dysfunction	75 (13.6%)	15 (16.1%)	0.627
Pupil diffusion			<0.001
No	248 (45%)	11 (11.8%)	
Unilateral	197 (35.8%)	40 (43%)	
Bilateral	106 (19.2%)	42 (45.2%)	
The way of DC			1.000
Unilateral	478 (86.8%)	81 (87.1%)	
Bilateral	73 (13.2%)	12 (12.9%)	
Method of operation			0.616
Direct decompression	493 (89.5%)	76 (81.7%)	
Progressive decompression	58 (10.5%)	17 (18.3%)	
Internal decompression	0 (0%)	8 (8.6%)	<0.001
Preoperative time (hour)	5.50 (4.50, 7.00)	5.30 (4.00, 6.50)	0.876
Operative time (hour)	2.83 (2.25, 3.58)	2.58 (2.08, 3.33)	0.335
Blood glucose	10.2 (8.70, 12.43)	11.3 (8.86, 14.77)	0.008
Death	107 (19.4%)	76 (81.7%)	<0.001

DC, decompressive craniectomy; GCS, Glasgow Coma Score; IOBB, intraoperative brain bulge.

On the CT images, brain contusion (79.6% vs. 33.8%), bilateral brain damage (41.9% vs. 2.4%), contralateral fracture (63.4% vs. 16.9%), degree of midline shift >15 mm (37.6% vs. 12.2%), disappear of the basal pool (30.1% vs. 3.1%), SDH (98.9% vs. 69.9%), and EDH (11.8% vs. 29.4%) were all associated with the development of IOBB. In particular, CT values of TH, SSS, HLTS, and DLTS were also strongly correlated with the occurrence of IOBB (Table 2).

**Table 2:** Differences between with no IOBB group and the IOBB group based on imaging features in the development cohort.

Characteristic	No IOBB (*n*=551)	IOBB (*n*=93)	*P*
Brain contusion	186 (33.8%)	74 (79.6%)	<0.001
Site of contusion			0.218
No	148 (26.9%)	28 (30.1%)	
Frontal lobe	195 (35.4%)	28 (30.1%)	
Temporal lobe	176 (31.9%)	30 (32.3%)	
Parietal lobe	6 (1.1%)	0 (0%)	
Occipital lobe	18 (3.3%)	7 (7.5%)	
Other	8 (1.5%)	0 (0%)	
Lesion side			<0.001
Unilateral	538 (97.6%)	54 (58.1%)	
Bilateral	13 (2.4%)	39 (41.9%)	
Contralateral fracture	93 (16.9%)	59 (63.4%)	<0.001
Midline shift			<0.001
＜5 mm	126 (22.9%)	15 (16.1%)	
5.1–10 mm	192 (34.8%)	21 (22.6%)	
10.1–15 mm	166 (30.1%)	22 (23.7%)	
＞15 mm	67 (12.2%)	35 (37.6%)	
Basal pool			<0.001
Normal	99 (18%)	7 (7.5%)	
Compression	435 (78.9%)	58 (62.4%)	
Disappear	17 (3.1%)	28 (30.1%)	
Multiple hematoma	392 (71.1%)	65 (69.9%)	0.903
IVH	28 (5.1%)	8 (8.6%)	0.261
SAH	413 (75%)	76 (81.7%)	0.200
SDH	385 (69.9%)	92 (98.9%)	<0.001
EDH	162 (29.4%)	11 (11.8%)	<0.001
CT value			
TH	50 (45, 54)	55 (50, 58)	<0.001
SSS	43 (36, 49)	49 (43, 52)	<0.001
HLTS	41 (34, 47)	43 (42, 49)	<0.001
DLTS	47 (44, 52)	57 (50, 60)	<0.001

CT, computed tomography; DLTS, diseased lateral transverse sinus; EDH, extradural hemorrhage; HLTS, healthy lateral transverse sinus; IOBB, intraoperative brain bulge; IVH, intraventricular hemorrhage; SAH, subarachnoid hemorrhage; SDH, subdural hemorrhage; SSS, superior sagittal sinus; TH, torcular herophili.

Since all 93 patients in the IOBB group had severe craniocerebral injuries (GCS: 3–8), only 78% of the 551 patients in the control group had severe craniocerebral injuries (GCS: 3–8). To prevent bias, 1:1 matching was performed using PSM based on GCS scores, and the results are presented in Supplementary Table S1 (Supplemental Digital Content 4, http://links.lww.com/JS9/B450). The probability of death (40.9% vs. 9.7%) and bilateral pupil dilation (22.6% vs. 10.8%) was significantly higher in patients in the IOBB group than in patients who did not develop IOBB. Patients who developed IOBB were younger than those who did not develop IOBB. The likelihood of post-traumatic coagulation dysfunction was similarly higher than in the control group (8.1% vs. 1.1%). On imaging, brain contusion (39.8% vs. 17.7%), bilateral brain injury (21.0% vs. 2.2%), contralateral fracture (31.7% vs. 10.2%), degree of midline shift >15 mm (18.8% vs. 7.5%), loss of basal pool (15.1% vs. 1.6%), SDH (49.5% vs. 36.6%), and EDH (5.9% vs. 14.0%) were all associated with the occurrence of IOBB. In particular, the CT values of TH, SSS, and DLTS were also strongly associated with the occurrence of IOBB.

### CT values of DLTS as a risk factor for IOBB

**Figure 1:**
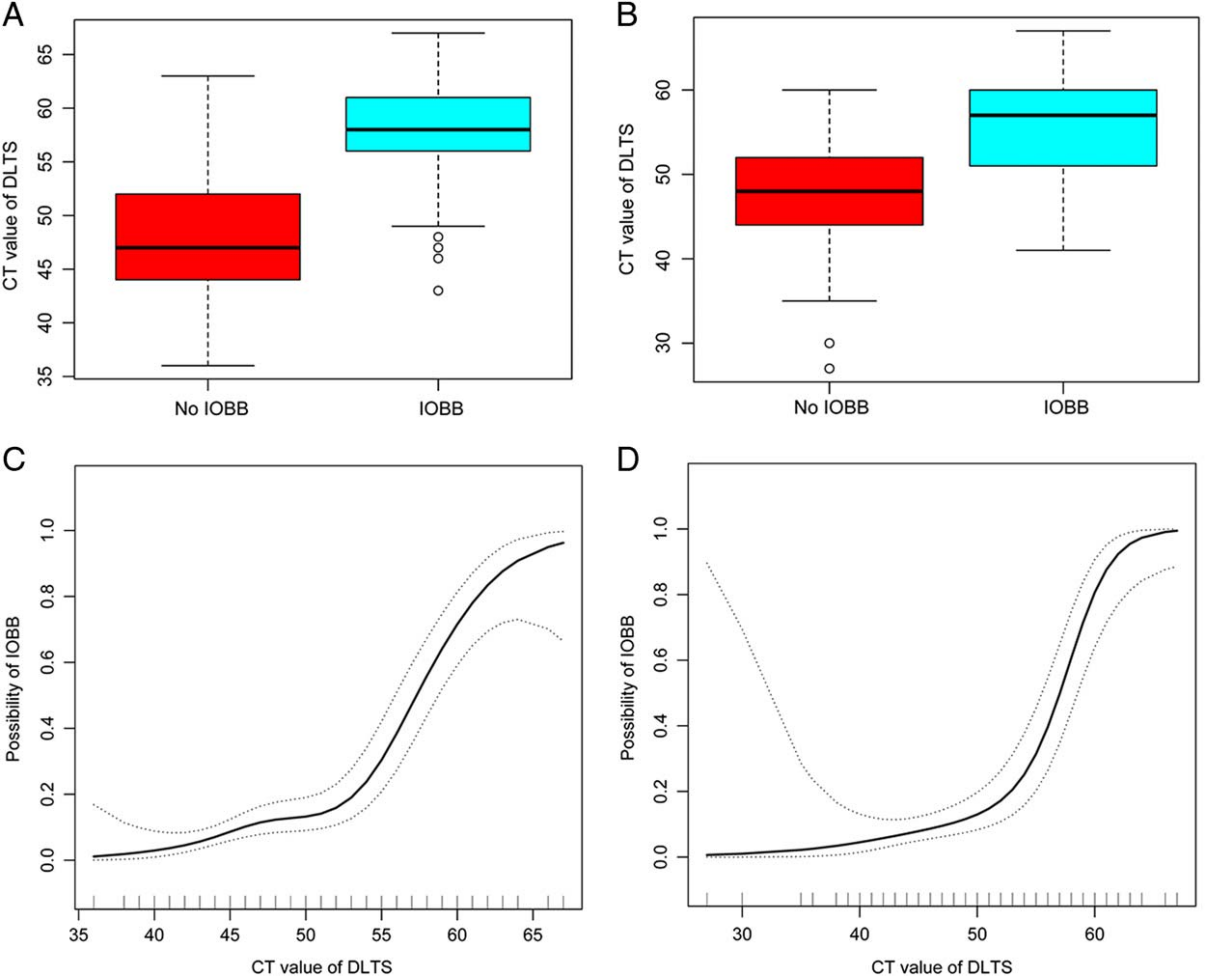
CT value of DLTS is positively associated with IOBB in patients with traumatic brain injury. (A) Box plots showing the CT value of DLTS distribution were different for the IOBB and non-IOBB groups in the development cohort. (B) The CT value of DLTS distribution was different for the IOBB and non-IOBB groups in the validation cohort. (C) Nonlinear relationship between the probability of IOBB and CT value of DLTS in the development cohort. (D) Nonlinear relationship between the probability of IOBB and CT value of DLTS in the validation cohort. CT, computed tomography; DLTS, diseased lateral transverse sinus; IOBB, intraoperative brain bulge.

**Figure 2:**
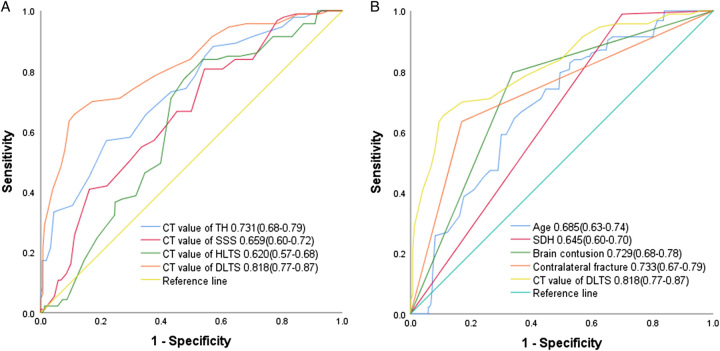
Comparison of areas under the receiver operating characteristic (ROC) curve for each index. (A) ROC curve analysis CT values of TH, SSS, HLTS, and DLTS for intraoperative brain bulge (IOBB) in the development cohort. (B) ROC curve analysis of other factors, such as age, SDH, brain contusion, and contralateral fracture, for IOBB in the development cohort. CT, computed tomography; DLTS, diseased lateral transverse sinus; HLTS, healthy lateral transverse sinus; SDH, subdural hemorrhage; SSS, superior sagittal sinus; TH, torcular herophili.

The overall distribution and median CT values of DLTS were higher in patients who developed IOBB than in those who did not develop IOBB (Fig. 1A). As the CT values of DLTS increased, the likelihood of patients developing IOBB increased. When the CT values ≥52, there was a linear relationship between the CT values of DLTS and the probability of IOBB (Fig. 1C). Univariate regression using the development cohort revealed an association between higher CT values of DLTS and IOBB (OR, 1.26; 95% CI, 1.20–1.32; Table 3). In multivariate regression, higher CT values of DLTS remained associated with IOBB after adjustment for other independent risk factors (OR, 1.22; 95% CI, 1.15–1.29; Table 3). The AUC of CT values of DLTS in predicting IOBB was 0.818 (95% CI, 0.77–0.87; Fig. 2A). The ROCs of CT values obtained were for TH (AUC=0.731), SSS (AUC=0.659), and HLTS (AUC=0.620) (Fig. 2A). Other indicators that predicted IOBB included age (AUC=0.685), SDH (AUC=0.645), brain contusion (AUC=0.729), and contralateral fracture (AUC=0.733; Fig. 2B).

**Table 3:** The univariate and multivariate regression analysis for predicting IOBB in the development cohort.

	Not adjusted		Adjustment	
Variable	OR (95% CI)	*P*	OR (95% CI)	*P*
Age	0.94 (0.92–0.96)	0.020	0.95 (0.91–0.99)	0.013
SDH	7.20 (2.17–23.89)	<0.001	9.76 (2.13–44.68)	<0.001
Contralateral fracture	8.55 (5.30–13.77)	<0.001	11.20 (5.65–22.20)	<0.001
Brain contusion	7.64 (4.48–13.04)	<0.001	3.57 (1.83–6.94)	<0.001
CT value of DLTS	1.26 (1.20–1.32)	<0.001	1.22 (1.15–1.29)	<0.001

CI, interval of confidence; DLTS, diseased lateral transverse sinus; IOBB, intraoperative brain bulge; OR, odds ratio; SDH, subdural hematoma.

### Predictive models for IOBB

**Figure 3:**
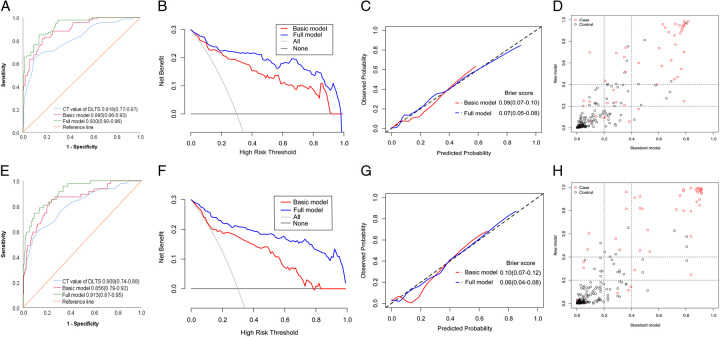
Establishment of the prediction model and demonstration of prediction ability. (A) Receiver operating characteristic (ROC) curve analysis of CT value of DLTS and basic and full models for intraoperative brain bulge (IOBB) in the development cohort. (B) Decision curve analysis (DCA) curve of basic and full models predicting IOBB. The full model shows a higher net benefit in the development cohort. (C) The consistency of the full model is better than that of the basic model in the development cohort. (D) Comparison of net classification improvement (NRI) and integrated discrimination improvement (IDI) between the two models in the development cohort. (E) ROC curve analysis of CT value of DLTS and basic and full models for IOBB in the validation cohort. (F) DCA curve of basic and full models predicting IOBB. The full model shows a higher net benefit in the validation cohort. (G) The consistency of the full model is better than that of the basic model in the validation cohort. (H) NRI and IDI were compared between the two models in the validation cohort. CT, computed tomography; DLTS, diseased lateral transverse sinus.

In the development cohort, two models (with and without CT values of DLTS) were developed to predict the likelihood of IOBB. Compared to the basic model based on traditional factors (age, SDH, brain contusion, and contralateral fracture), adding CT values of DLTS to the model (full model) significantly improved sensitivity (82.18% vs. 71.50%), specificity (92.31% vs. 84.45%) and overall performance (AUC, 0.930 [95% CI, 0.90–0.96] vs. 0.895 [95% CI, 0.86–0.93]) (Fig. 3A). DCA suggested that adding CT values of DLTS to the full model yielded a net benefit over the basic model (Fig. 3B). The calibration curves suggested that the full model predictions were in better agreement with observations than the basic model, and the Brier score of the full model was smaller than that of the basic model (Fig. 3C). The corresponding median NRI and IDI values were 0.185 (95% CI, 0.07–0.30; *P*=0.002) and 0.114 (95% CI, 0.07–0.15; *P*<0.001) (Fig. 3D).

### External validation

The demographic and clinical characteristics of the basic clinical data of the validation cohort of 293 patients are shown in Supplementary Table S2 (Supplemental Digital Content 5, http://links.lww.com/JS9/B451), while the metrics on CT images are displayed in Supplementary Table S3 (Supplemental Digital Content 6, http://links.lww.com/JS9/B452). In the validation cohort, the overall distribution and median CT values of DLTS were higher in patients who developed IOBB than in patients who did not develop IOBB (Fig. 1B). As the CT values of DLTS increased, the likelihood of patients developing IOBB increased (Fig. 1D). The AUC for CT values of DLTS predicting IOBB was 0.808 (95% CI, 0.74–0.88; Fig. 3E).

Univariate regression using the validation cohort revealed an association between higher CT values of DLTS and IOBB (OR, 1.28; 95% CI, 1.15–1.42; Supplementary Table S4, Supplemental Digital Content 7, http://links.lww.com/JS9/B453). In multivariate regression, higher CT values of DLTS remained associated with IOBB after adjustment for other independent risk factors (OR, 1.26; 95% CI, 1.15–1.39; Supplementary Table S4, Supplemental Digital Content 7, http://links.lww.com/JS9/B453). The AUC of the basic model was 0.859 (95% CI, 0.82–0.90) with a sensitivity of 70.99% and a specificity of 84.36%. Adding CT values of DLTS to the full model improved sensitivity (84.36%), specificity (90.27%), and overall performance (AUC, 0.913 [95% CI, 0.87–0.95]; Fig. 3E). DCA suggested that adding CT values of DLTS to the full model yielded a net benefit over the basic model (Fig. 3F). The calibration curves showed good agreement between predictions and observations for the full model (Fig. 3G). The corresponding median NRI and IDI values were 0.171 (95% CI, 0.03–0.31; *P*=0.017) and 0.211 (95% CI, 0.14–0.29; *P*<0.001) (Fig. 3H).

### Comparison of machine learning model and logical model

**Figure 4:**
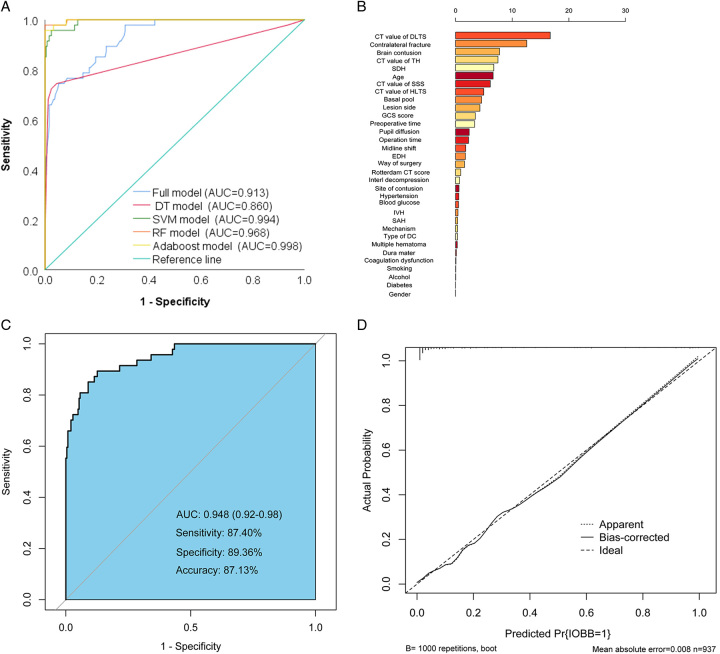
Logistic regression analysis model and comparison of different machine learning models. (A) Receiver operating characteristic (ROC) curve analysis of the full model and different machine learning models. (B) Ranking of the importance of the factors in the AdaBoost algorithm. (C) The ROC curve of the new model was constructed by combining logistic regression analysis and machine learning models. (D) Calibration curve analysis of the new model. AUC, area under curve; CT, computerized tomography; DC, decompressive craniectomy; DLTS, diseased lateral transverse sinus; DT, decision tree; EDH, extradural hemorrhage; GCS, Glasgow Coma Score; HLTS, healthy lateral transverse sinus; IOBB, intraoperative brain bulge; IVH, intraventricular hemorrhage; SAH, subarachnoid hemorrhage; SSS, superior sagittal sinus; SVM, support vector machine; TH, torcular herophili.

Several machine learning models, including the DT, RF, SVM, and AdaBoost models, were constructed for comparison with the logistic regression analysis model (full model) in the validation cohort (Table 4). The AdaBoost model had higher sensitivity (95.74%), specificity (100%), accuracy (99.32%), and AUC (0.998 [95% CI, 0.99–1.00]) than other models (Fig. 4A). The Delong test suggests that the ROC curves of several machine learning models are statistically different when compared to the full model (Table 4). The top six factors in the importance ranking of each factor in the AdaBoost model contained all the variables in the full model (age, SDH, brain contusion, contralateral fracture, and CT values of DLTS). However, it is worth noting that the CT values for TH ranked fourth in importance, surpassing age and SDH (Fig. 4B). Therefore, we combined the CT values of TH and the full model and reapplied the AdaBoost algorithm to derive a new ROC that had an AUC of 0.948 (95% CI, 0.92–0.98), excellent specificity (89.36%), sensitivity (87.40%), and accuracy (87.13%) (Fig. 4C). The calibration curves showed good agreement between predictions and observations for the full model (Fig. 4D).

**Table 4:** Comparison of the effectiveness of logistic regression analysis model (full model) and other models from machine learning in the validation cohort.

Model	AUC (95% CI)	Specificity (%)	Sensitivity (%)	Accuracy (%)	*P*
Full model	0.913 (0.87–0.95)	94.72	74.47	91.47	Reference
DT model	0.860 (0.78–0.94)	95.53	73.52	92.15	0.039
SVM model	0.994 (0.99–1.00)	97.56	95.74	97.27	<0.001
RF model	0.968 (0.93–0.99)	100	97.87	98.98	<0.001
AdaBoost model	0.998 (0.99–1.00)	100	95.74	99.32	<0.001

AUC, area under curve; CI, confidence interval; DT, decision tree; RF, random forest; SVM, support vector machine.

### Construction of the nomogram

**Figure 5:**
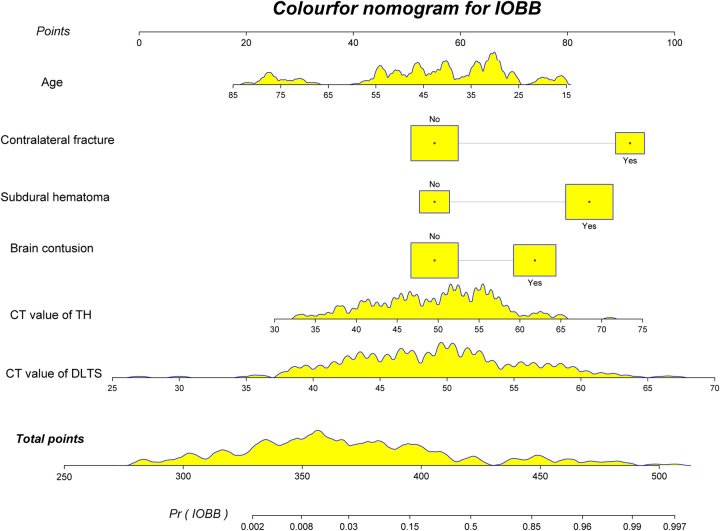
Nomogram, constructed from the new model, to predict IOBB. Nomogram according to the various influencing factors on outcome variables in the model. The impact (the size of the regression coefficient for each level of each value of factors) is assigned points; then, the scores are added to get the total score, which determines the individual event prediction probability. CT, computerized tomography; DLTS, diseased lateral transverse sinus; IOBB, intraoperative brain bulge; TH, torcular herophili.

To facilitate clinical application, a novel nomogram combining the full model and CT values of TH was created for predicting the occurrence of IOBB (Fig. 5). In the nomogram, the score includes the single item score (points in the figure), which represents the single item score corresponding to each variable, and the total points represent combined with the single item score corresponding to all variables. The total score for each patient corresponds to the probability of an unfavorable prognosis.

**Figure 6:**
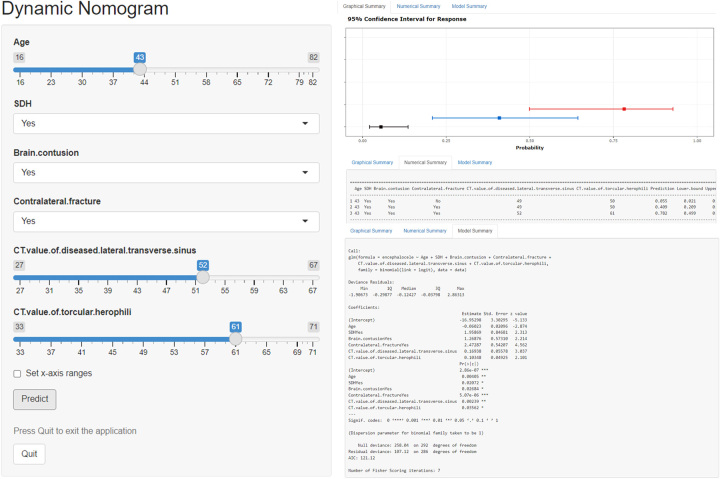
Shinyapps.io platform to predict the intraoperative brain bulge of patients with traumatic brain injury (https://dzzhuang.shinyapps.io/dynnomapp/). Users can submit values for the six features into the corresponding text box of the web page through the computer or mobile phone for calculation. Once the output of the sample has been calculated, the results page will display the probability of IOBB, the 95% confidence interval, and the parameters of the model. CT, computerized tomography; SDH, subdural hemorrhage.

To facilitate the application of the prediction model, we upgraded the nomogram to a dynamic nomogram and uploaded it to the shinyapps.io platform (https://dzzhuang.shinyapps.io/dynnomapp/) to predict the occurrence of IOBB. Users can submit the six features (age, SDH, brain contusion, contralateral fracture, CT values of TH, and CT values of DLTS) to the corresponding text box of the web page for calculation through the computer or mobile phone (Fig. 6). After calculating the output of the sample, the results page will display the probability of IOBB, the 95% confidence interval, and the parameters of the model.

## Discussion

The results of this study showed that the occurrence of IOBB in patients with TBI is strongly associated with the CT value of DLTS. As the CT value of DLTS increases, the likelihood of IOBB occurs. In multifactorial regression analysis using data from the development cohort, IOBB was also independently associated with other risk factors, such as age, SDH, contralateral fracture, and brain contusion. Both our study and previous studies suggest that younger age is an independent predictor for the development of diffuse brain swelling in patients with SDH^32^. This is due to the large intracranial space after brain atrophy in older adults, which allows for less severe compression of brain tissue by hematoma, lower immunoinflammatory response, and reduced secondary injury than in younger adults. Studies of biochemical, cellular, and molecular responses to TBI in juvenile and adult animals have found that juvenile animals show a more pronounced inflammatory response and greater neurodegeneration after injury^33^. Previous studies have shown that SDH is a significant indicator associated with IOBB^5^. Acute SDH exacerbates cerebral blood flow disorder and promotes the development of IOBB in patients with severe TBI. In this study, SDH was also an independent predictor of IOBB (OR, 7.20; 95% CI, 2.17–23.89; AUC, 0.645), confirming the validity of the study. Contralateral injuries are common injuries in patients with TBI. In particular, when SDH is combined with a contralateral fracture, there is a higher likelihood of EDH at the fracture and consequent IOBB during DC surgery^34,35^. The results of this study also confirm a strong correlation between contralateral fracture and IOBB (OR, 8.55; 95% CI, 5.30–13.77; AUC, 0.733). Our study showed that brain contusion is highly associated with the occurrence of IOBB (OR, 7.64; 95% CI, 4.48–13.04; AUC, 0.729). Cerebral edema develops around the site of brain contusion over a period of hours to days after TBI and gradually progresses to a peak^36^. Microcirculatory impairment due to brain contusion is the main pathophysiological change after TBI, is generally considered to be a major link leading to secondary injury, such as cerebral edema, and is one of the possible mechanisms of IOBB^37^.

The current research on IOBB has evolved from sporadic case reports to several recent retrospective studies on the risk factors for IOBB^38–40^. The occurrence of IOBB in TBI patients during DC surgery often catches surgeons off guard and suggests a poor prognosis of disability and death. How to anticipate IOBB before surgery has become a greater concern for surgeons. Our study not only explores the risk factors associated with the occurrence of IOBB in DC surgery but also constructs a prediction model to predict the likelihood of IOBB. In the construction of the model, not only the traditional approach of logistic regression analysis was used, but also a machine learning model was introduced for comparison^41^. Although the machine learning models, such as AdaBoost, had significantly higher AUCs than the logistic regression model and showed excellent specificity, sensitivity, and accuracy (Table 4), the AdaBoost model required the inclusion of all collected risk factors, whereas the logistic regression model included only five independent risk factors. It is impractical to include all risk factors in order to make a prediction model clinically useful. Therefore, this study analyzed the importance ranking of the relevant risk factors in the AdaBoost model (Fig. 4B) and compared it with the independent risk factors in the logistic regression model^42^. By combining the two models with logistic regression and machine learning, the risk factors were reincorporated to construct a more efficient and sensitive prediction model (Figs 5, 6). However, in this study, machine learning modeling was a complementary tool rather than a major component and had little impact on the eventual development of predictive models applicable to clinicians. Therefore, we did not refine the validation of the machine learning models much, which may be a flaw in the article.

A total of six variables involving age, SDH, brain contusion, contralateral fracture, and the CT values of TH and DLTS were included in the final prediction model. The CT values of TH and DLTS are actually density values of the venous sinuses, expressed as HU, representing the state of the cerebral venous sinuses. Previous studies have shown that HU values are closely related to blood viscosity^43^. The higher the HU value, the more viscous the blood is and the slower the blood flow. When HU>70, blood may even clot and thrombosis may occur^44^. In our study, none of the patients had DLTS values >70. This suggests that the etiology of IOBB may not be transverse sinus thrombosis, but may be related to overall blood flow to some extent. Of course, it is not known whether hematomas around the venous sinuses when they are injured can cause thrombosis in subsequent development or have an effect on the measurement of CT values. When a patient with TBI requires DC surgery, it often suggests a large subdural hematoma with significant occupancy effects^45,46^. After the subdural hematoma compresses the cerebral cortex, the cortical and bridging veins are compressed, and blood flow back into the transverse sinus is reduced. During DC surgery, after the relief of the occupancy effect, the arterial blood flow recovers rapidly while the venous blood flow recovers slowly, causing the brain tissue to fill with blood and expand outward, which may be one of the mechanisms for IOBB formation^47–49^.

The return of blood from the arteries to the veins needs to pass through microcirculation, which may be a new mechanism and therapeutic target for IOBB. Previous literature has shown that in mouse models, subdural hematomas compressing the posterior cortical microcirculation gradually form thrombi, causing cellular ischemic damage and leading to further neuronal damage and astrocyte swelling^50,51^. In the TBI mouse model, rolling of leukocytes on the cerebrovascular endothelium was observed in both small arteries and small veins, while leukocyte–platelet aggregates were found only in small veins, and microthrombi obstructed up to 70% of small veins and 33% of small arteries^52^. In addition, inflammatory responses play an important role in the development of microcirculatory disorders. Microcirculatory responses to inflammation include impaired vasodilatation, reduced capillary perfusion, leukocyte and platelet adhesion, activation of the coagulation cascade, enhanced thrombosis, increased vascular permeability, and increased rates of blood and lymphatic vessel proliferation^53^. Activation products and chemical mediators released from endothelial, pericytes, and inflammatory cells act through different well-characterized signaling pathways to induce changes in microvascular function^53^. Notably, IOBB occurs in only a small percentage of patients. This seems to be a matter of balance between microcirculation and macroscopic cerebral blood flow and deserves further exploration.

Although we tried to make this study more complete and meaningful, there are still some limitations. First, as a retrospective study, the results were subject to selection bias and missing information. The use of two independent cohorts may help to reduce bias, but future prospective multicenter cohort studies are still needed for validation. Second, microcirculatory impairment after trauma may be associated with an inflammatory response, as found in previous studies, but this part of the inflammatory index was missing in our study. Third, the prediction model in this study is only applicable to TBI patients who require DC surgery and is not applicable to all TBI patients. Finally, the outcome variables in this study were collected from surgical records, which may have missed some cases, and subsequent studies should consider more appropriate prospective studies.

## Conclusion

This study suggests that CT values of DLTS are highly correlated with the occurrence of IOBB in DC surgery of patients with TBI and is a new imaging predictor. Combining the advantages of logistic regression analysis and machine learning models, one can create more effective IOBB prediction models for clinicians to use.

## Ethical approval

The study was approved by the Fuzong Clinical Medical College of Fujian Medical University (No: 2023-045) and the First Affiliated Hospital of Shantou University Medical College (No: B-2022-004).

## Patient consent

Written informed consent was obtained from the patient for the publication of this case report and accompanying images. A copy of the written consent is available for review by the Editor-in-Chief of this journal on request.

## Consent for publication

All claims expressed in this article are solely those of the authors and do not necessarily represent those of their affiliated organizations or those of the publisher, the editors, and the reviewers.

## Sources of funding

This work was supported by the Natural Science Foundation of Guangdong Province (2022A1515012144, 2023A1515012055), the Natural Science Foundation of Hunan Province (2021JJ40929), and the Fujian Provincial Science and Technology Innovation Joint Fund (2019Y9045).

## Author contribution

D.Z., T.L., K.L., W.C., and S.W.: were responsible for the study concept and design; D.Z., H.X., X.C., and J.S.: were responsible for the analysis and interpretation of data; D.Z., T.L., and W.C.: were responsible for the drafting of the manuscript; X.L. and J.S.: were responsible for data collection; D.Z., W.C., and S.W.: were responsible for the critical revision of the manuscript for important intellectual content.

## Conflicts of interest disclosure

The authors declare that the research was conducted in the absence of any commercial or financial relationships that could be construed as a potential conflict of interest.

## Research registration unique identifying number (UIN)

1. Name of the registry: Researchregistry.com (Weiqiang Chen).
2. Unique identifying number or registration ID: researchregistry9159.
3. Hyperlink to your specific registration (must be publicly accessible and will be checked): https://www.researchregistry.com/registernow#home/registrationdetails/648f326ca155e20028673117/.

## Guarantor

Dongzhou Zhuang, Tian Li, Kangsheng Li, Weiqiang Chen, and Shousen Wang.

## Data availability statement

The data that support the findings of this study are available from the corresponding author upon reasonable request.

## Provenance and peer review

Not applicable.

## Supplementary Material
