# Supplementary figures and images for: A dynamic nomogram for predicting intraoperative brain bulge during decompressive craniectomy in patients with traumatic brain injury: a retrospective study
Source: Int J Surg. 2023 Dec 2;110(2):909–20. doi: 10.1097/JS9.0000000000000892 (PMC10871569; doi:10.1097/JS9.0000000000000892)

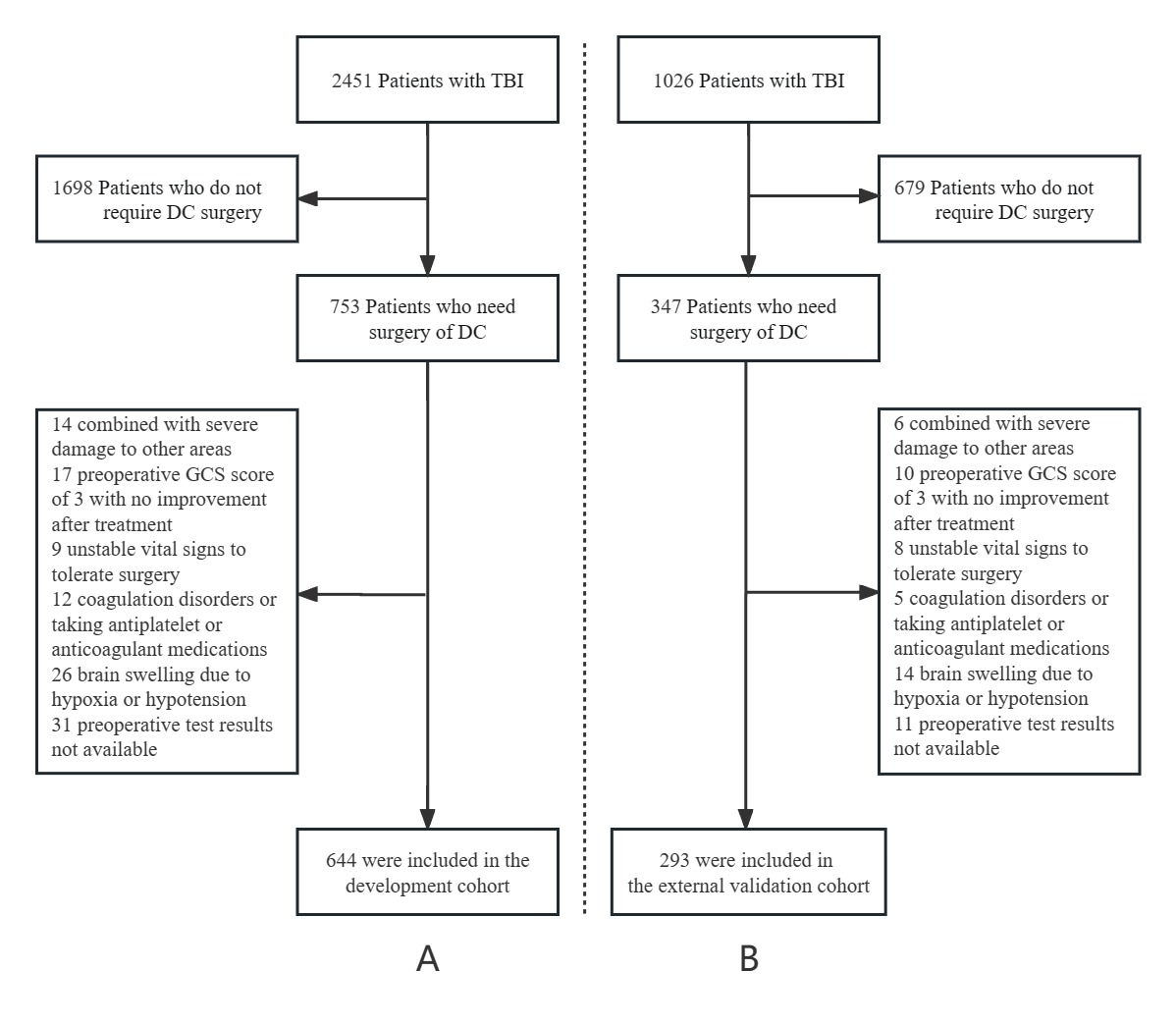

Supplement: Supplementary file 2 [file js9-110-0909-s002.jpg]

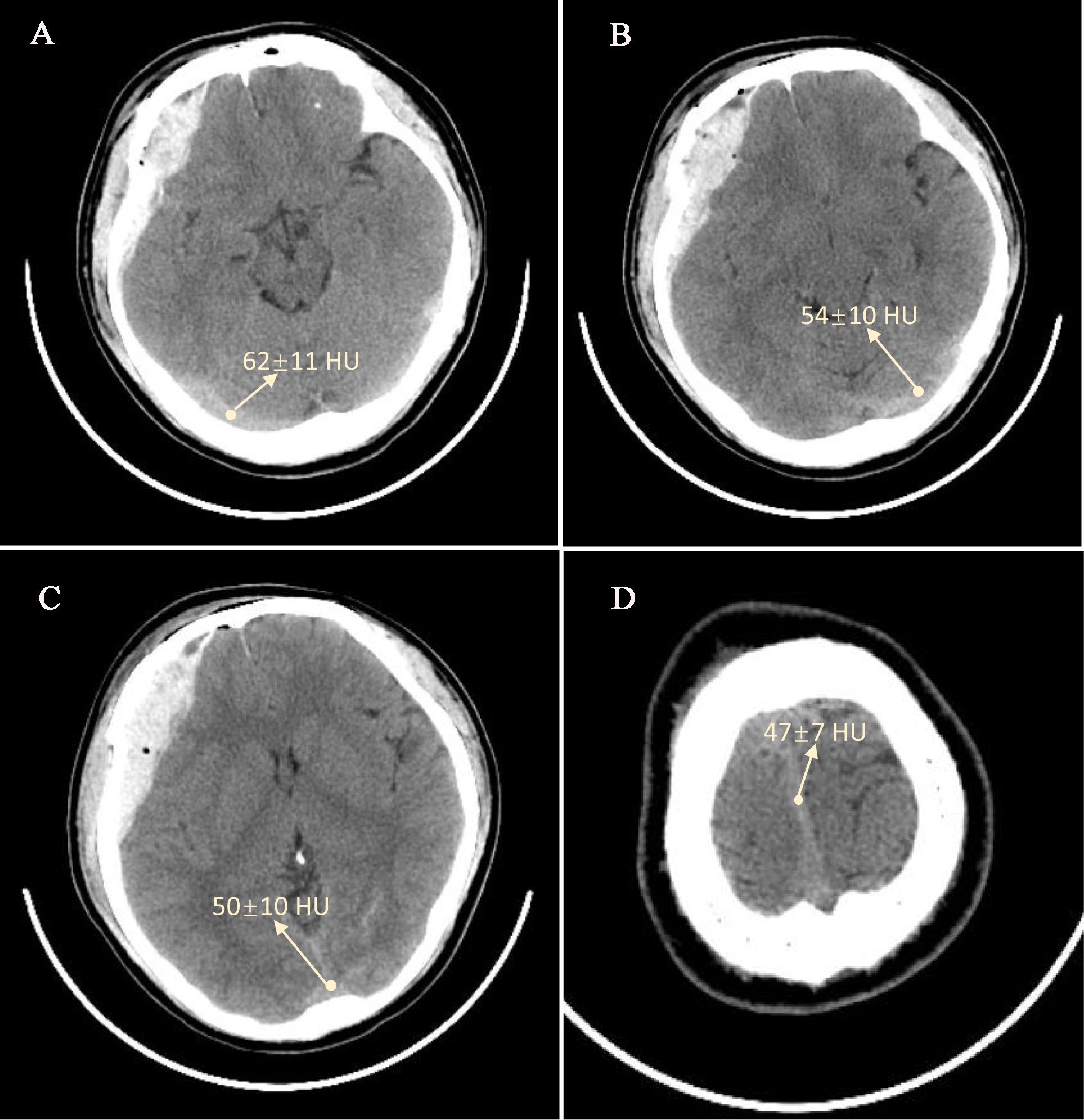

Supplement: Supplementary file 3 [file js9-110-0909-s003.jpg]
