# Supplementary material for: A dynamic nomogram for predicting intraoperative brain bulge during decompressive craniectomy in patients with traumatic brain injury: a retrospective study
Source: Int J Surg. 2023 Dec 2;110(2):909–20. doi: 10.1097/JS9.0000000000000892 (PMC10871569; doi:10.1097/JS9.0000000000000892)
Supplement: Supplementary file 4 [file js9-110-0909-s004.docx]

Table S1. Differences between with no IOBB group and the IOBB group in the development cohort after propensity score matching.

| Characteristic | IOBB (n=93) | No IOBB (n=93) | *P*-value |
| --- | --- | --- | --- |
| Gender (male) | 74 (39.8%) | 83 (44.6%) | 0.069 |
| Age | 48 (31, 60) | 55 (48, 61) | < 0.001 |
| Mechanism of injury |  |  | 0.068 |
| Car accident | 68 (36.6%) | 53 (28.5%) |  |
| High fall injury | 20 (10.8%) | 33 (17.7%) |  |
| Other | 5 (2.7%) | 7 (3.8%) |  |
| Coagulation dysfunction | 15 (8.1%) | 2 (1.1%) | < 0.001 |
| Pupil diffusion |  |  | < 0.001 |
| No | 11 (5.9%) | 32 (17.2%) |  |
| Unilateral | 40 (21.5%) | 41 (22%) |  |
| Bilateral | 42 (22.6%) | 20 (10.8%) |  |
| The way of DC |  |  | 1.000 |
| Unilateral | 81 (43.5%) | 81 (43.5%) |  |
| Bilateral | 12 (6.5%) | 12 (6.5%) |  |
| Method of operation |  |  | 0.312 |
| Direct decompression | 81 (43.5%) | 76 (40.9%) |  |
| Progressive decompression | 12 (6.5%) | 17 (9.1%) |  |
| Internal decompression | 8 (8.6%) | 0 (0%) | < 0.001 |
| Preoperative time (h) | 5.50 (4.30, 6.70) | 5.75 (4.50, 7.00) | 0.685 |
| Operative time (h) | 2.58 (2.08, 3.33) | 2.75 (2.08, 3.67) | 0.589 |
| Blood glucose | 11.30 (8.86, 14.77) | 10.70 (9.48, 12.43) | 0.052 |
| Brain contusion | 74 (39.8%) | 33 (17.7%) | < 0.001 |
| Site of contusion |  |  | 0.051 |
| No | 28 (30.1%) | 19 (20.4%) |  |
| Frontal lobe | 28 (30.1%) | 47 (50.5%) |  |
| Temporal lobe | 30 (32.3%) | 21 (22.5%) |  |
| Parietal lobe | 0 (0%) | 1 (1.08%) |  |
| Occipital lobe | 7 (7.5%) | 5 (5.38%) |  |
| Lesion side |  |  | < 0.001 |
| Unilateral | 54 (29%) | 89 (47.8%) |  |
| Bilateral | 39 (21%) | 4 (2.2%) |  |
| Contralateral fracture | 59 (31.7%) | 19 (10.2%) | < 0.001 |
| Midline shift |  |  | 0.002 |
| ＜5mm | 15 (8.1%) | 20 (10.8%) |  |
| 5.1-10mm | 21 (11.3%) | 38 (20.4%) |  |
| 10.1-15mm | 22 (11.8%) | 21 (11.3%) |  |
| ＞15mm | 35 (18.8%) | 14 (7.5%) |  |
| Basal pool |  |  | < 0.001 |
| Normal | 7 (3.8%) | 9 (4.8%) |  |
| Compression | 58 (31.2%) | 81 (43.5%) |  |
| Disappear | 28 (15.1%) | 3 (1.6%) |  |
| Multiple hematoma | 65 (34.9%) | 70 (37.6%) | 0.411 |
| IVH | 8 (4.3%) | 4 (2.2%) | 0.233 |
| SAH | 76 (40.9%) | 69 (37.1%) | 0.216 |
| SDH | 92 (49.5%) | 68 (36.6%) | < 0.001 |
| EDH | 11 (5.9%) | 26 (14%) | 0.006 |
| CT value |  |  |  |
| TH | 55 (50, 58) | 51 (46, 55) | < 0.001 |
| SSS | 49 (43, 52) | 45 (36, 50) | 0.004 |
| HLTS | 43 (42, 49) | 44 (35, 51) | 0.793 |
| DLTS | 57 (50, 60) | 49 (44, 52) | < 0.001 |
| Death | 76 (40.9%) | 18 (9.7%) | < 0.001 |

Abbreviations: IOBB, intraoperative brain bulge; GCS, Glasgow Coma Score; DC, decompressive craniectomy; IVH, intraventricular haemorrhage; SAH, subarachnoid haemorrhage; SDH, subdural haemorrhage; EDH, extradural haemorrhage; CT, computerized tomography; TH, torcular herophili; SSS, superior sagittal sinus; HLTS, healthy lateral transverse sinus; DLTS, diseased lateral transverse sinus.
