# Supplementary material for: A dynamic nomogram for predicting intraoperative brain bulge during decompressive craniectomy in patients with traumatic brain injury: a retrospective study
Source: Int J Surg. 2023 Dec 2;110(2):909–20. doi: 10.1097/JS9.0000000000000892 (PMC10871569; doi:10.1097/JS9.0000000000000892)
Supplement: Supplementary file 5 [file js9-110-0909-s005.docx]

Table S2. Differences between with no IOBB group and the IOBB group in the validation cohort.

| Characteristic | No IOBB (n=246) | | IOBB (n=47) | | *P*-value | |
| --- | --- | --- | --- | --- | --- | --- |
| Gender (male) | 194 (78.9%) | | 37 (78.7%) | | 1.000 | |
| Age | 57 (39.5, 65.5) | | 48 (35, 59) | | 0.045 | |
| GCS grade |  | |  | | 0.002 | |
| Mild (13–15 score) | 14 (4.8%) | | 0 (0%) | |  | |
| Moderate (9–12 score) | 33 (11.3%) | | 0 (0%) | |  | |
| Severe (3–8 score) | 199 (67.9%) | | 47 (16%) | |  | |
| Mechanism of injury |  | |  | | 0.053 | |
| Car accident | 150 (61.0%) | | 37 (78.7%) | |  | |
| High fall injury | 83 (33.7%) | | 8 (17%) | |  | |
| Other | 13 (5.3%) | | 2 (4.3%) | |  | |
| Coagulation dysfunction | 35 (14.2%) | | 10 (21.3%) | | 0.314 | |
| Pupil diffusion |  | |  | | < 0.001 | |
| No | 107 (43.5%) | | 6 (12.8%) | |  | |
| Unilateral | 100 (40.7%) | | 17 (36.2%) | |  | |
| Bilateral | 39 (15.9%) | | 24 (51.1%) | |  | |
| The way of DC |  | |  | | 0.378 | |
| Unilateral | 214 (87.0%) | | 38 (80.9%) | |  | |
| Bilateral | 32 (13.0%) | | 9 (19.1%) | |  | |
| Method of operation | |  | |  | | 0.438 |
| Direct decompression | | 218 (89.0%) | | 44 (93.6%) | |  |
| Progressive decompression | | 27 (11.0%) | | 3 (6.4%) | |  |
| Internal decompression | 0 (0%) | | 3 (6.4%) | | 0.004 | |
| Preoperative time (h) | 5.75 (4.50, 8.00) | | 5.00 (4.00, 6.50) | | 0.151 | |
| Operative time (h) | 2.79 (2.25, 3.50) | | 2.58 (2.08, 3.42) | | 0.618 | |
| Blood glucose | 10.15 (8.60, 12.40) | | 11 (9.05, 14.63) | | 0.089 | |
| Death | 51 (20.7%) | | 33 (70.2%) | | < 0.001 | |

Abbreviations: IOBB, intraoperative brain bulge; GCS, Glasgow Coma Score; DC, decompressive craniectomy
