# Supplementary material for: A dynamic nomogram for predicting intraoperative brain bulge during decompressive craniectomy in patients with traumatic brain injury: a retrospective study
Source: Int J Surg. 2023 Dec 2;110(2):909–20. doi: 10.1097/JS9.0000000000000892 (PMC10871569; doi:10.1097/JS9.0000000000000892)
Supplement: Supplementary file 6 [file js9-110-0909-s006.docx]

Table S3. Differences between with no IOBB group and the IOBB group based on imaging features in the validation cohort.

| Characteristic | No IOBB (n=246) | IOBB (n=47) | *P*-value |
| --- | --- | --- | --- |
| Brain contusion | 90 (36.6%) | 40 (85.1%) | < 0.001 |
| Site of contusion |  |  | 0.066 |
| No | 68 (27.6%) | 7 (14.9%) |  |
| Frontal lobe | 94 (38.2%) | 8 (17.0%) |  |
| Temporal lobe | 69 (28.0%) | 16 (34.0%) |  |
| Parietal lobe | 3 (1.2%) | 6 (12.8%) |  |
| Occipital lobe | 10 (4.1%) | 4 (8.5%) |  |
| Other | 2 (0.8%) | 6 (12.8%) |  |
| Lesion side |  |  | < 0.001 |
| Unilateral | 241 (82.3%) | 30 (10.2%) |  |
| Bilateral | 5 (1.7%) | 17 (5.8%) |  |
| Contralateral fracture | 45 (18.3%) | 34 (72.3%) | < 0.001 |
| Midline shift |  |  | 0.006 |
| ＜5mm | 60 (24.4%) | 6 (12.8%) |  |
| 5.1-10mm | 79 (32.1%) | 10 (21.3%) |  |
| 10.1-15mm | 74 (30.1%) | 16 (34.0%) |  |
| ＞15mm | 33 (13.4%) | 15 (31.9%) |  |
| Basal pool |  |  | < 0.001 |
| Normal | 47 (16.0%) | 3 (1.0%) |  |
| Compression | 183 (62.5%) | 25 (8.5%) |  |
| Disappear | 16 (5.5%) | 19 (6.5%) |  |
| Multiple hematoma | 176 (71.5%) | 28 (59.6%) | 0.144 |
| IVH | 13 (5.3%) | 4 (8.5%) | 0.491 |
| SAH | 186 (75.6%) | 41 (87.2%) | 0.119 |
| SDH | 186 (75.6%) | 41 (87.2%) | < 0.001 |
| EDH | 74 (30.1%) | 6 (12.8%) | 0.024 |
| CT value |  |  |  |
| TH | 49 (43, 54) | 57 (52, 61.5) | < 0.001 |
| SSS | 43 (35, 49) | 48 (43, 51) | < 0.001 |
| HLTS | 41 (34, 47.75) | 45 (42, 50.5) | 0.001 |
| DLTS | 48 (44, 52) | 57 (51, 60) | < 0.001 |

Abbreviations: IOBB, intraoperative brain bulge; IVH, intraventricular haemorrhage; SAH, subarachnoid haemorrhage; SDH, subdural haemorrhage; EDH, extradural haemorrhage; CT, computerized tomography; TH, torcular herophili; SSS, superior sagittal sinus; HLTS, healthy lateral transverse sinus; DLTS, diseased lateral transverse sinus.
