# Supplementary material for: A dynamic nomogram for predicting intraoperative brain bulge during decompressive craniectomy in patients with traumatic brain injury: a retrospective study
Source: Int J Surg. 2023 Dec 2;110(2):909–20. doi: 10.1097/JS9.0000000000000892 (PMC10871569; doi:10.1097/JS9.0000000000000892)
Supplement: Supplementary file 7 [file js9-110-0909-s007.docx]

Table S4. The Univariate and multivariate regression analysis for predicting IOBB in the validation cohort.

| Variable | Not adjusted | | | | Adjustment | | | | |
| --- | --- | --- | --- | --- | --- | --- | --- | --- | --- |
|  | OR (95% CI) | | *P-* Value | | | OR (95% CI) | *P-* Value | | |
| Age | | 0.93 (0.86, 0.99) | | 0.030 | 0.96 (0.93, 0.99) | | | 0.006 | |
| SDH | | 19.25 (3.79, 97.72) | | <0.001 | 13.74 (2.96, 63.77) | | | | 0.001 |
| Contralateral fracture | | 11.94 (4.19, 33.96) | | <0.001 | 13.85 (5.05, 37.97) | | | | <0.001 |
| Brain contusion | | 6.69 (2.02, 22.12) | | 0.002 | 8.24 (2.74,24.83) | | | | <0.001 |
| CT value of DLTS | | 1.28 (1.15, 1.42) | | <0.001 | 1.26 (1.15, 1.39) | | | | <0.001 |

Abbreviations: IOBB, intraoperative brain bulge; SDH, subdural hematoma; OR, odds ratio; CI, interval of confidence; DLTS, diseased lateral transverse sinus.
